# Reduced Risk of Hospitalization With Stronger Community Belonging Among Aging Canadians Living With Diabetes: Findings From Linked Survey and Administrative Data

**DOI:** 10.3389/fpubh.2021.670082

**Published:** 2021-05-14

**Authors:** Neeru Gupta, Zihao Sheng

**Affiliations:** ^1^Department of Sociology, University of New Brunswick, Fredericton, NB, Canada; ^2^Department of Economics, Dalhousie University, Halifax, NS, Canada

**Keywords:** hospitalization - statistics and numerical data, diabetes mellitus, data linkage analysis, community belonging, aging adults

## Abstract

**Background:** Social isolation has been identified as a substantial health concern in aging populations, associated with adverse chronic disease outcomes and health inequalities; however, little is known about the interconnections between social capital, diabetes management, and hospital burdens. This study aimed to assess the role of community belonging with the risk of potentially avoidable hospitalization among aging adults living with diabetes in Canada.

**Methods:** The study leveraged a novel resource available through Statistics Canada's Social Data Linkage Environment: the Canadian Community Health Survey linked to administrative health records from the hospital Discharge Abstract Database. A population-representative sample of 13,580 community-dwelling adults aged 45 and over with diabetes was identified. Multiple logistic regression was used to assess the association of individuals' sense of community belonging with the risk of diabetes-related hospitalization over the period 2006–2012.

**Results:** Most (69.9%) adults with diabetes reported a strong sense of belonging to their local community. Those who reported weak community belonging were significantly more likely to have been hospitalized for diabetes (χ^2^ = 13.82; *p* < 0.05). The association between weak community attachment and increased risk of diabetes hospitalization remained significant [adjusted OR: 1.80 (95%CI: 1.12–2.90)] after controlling for age, education, and other sociodemographic and behavioral factors.

**Conclusion:** The COVID-19 pandemic has resurfaced attention to the need to better address social capital and diabetes care in public health strategies. While the causal pathways are unclear, this national study highlighted that deficits in social attachments may place adults with diabetes at greater risk of acute complications leading to hospitalization.

## Introduction

The strength of social bonds, in terms of both an individual resource as well as a social resource, has been widely theorized as a determinant of population health, including inequalities in outcomes related to diabetes mellitus and other chronic ambulatory care sensitive conditions prevalent in aging societies (1–6). While the literature lacks consensus on the explanatory pathways between social capital and health (1, 7), it has been reasoned that beneficial social connections foster the ability of individuals and groups to access public health service interventions, avoid health risks, and adopt health-enhancing and health-protective behaviors (8, 9). On the other hand, societal disinvestment in social capital has been hypothesized as linked to (unmeasured) societal tolerance of health inequalities and, in turn, higher rates of deleterious health-related behaviors and adverse health outcomes (7). The COVID-19 pandemic, which is argued to have brought significant disruptions in social capital with physical distancing protocols, has resurfaced attention to the need to better address community bonding and belonging as part of public health response and recovery plans (10, 11).

Beyond biological age and genetic predispositions, poor social support has been found to significantly increase the risk of incident type 2 diabetes (12), whereas stronger community belonging has been shown to exercise a positive relationship with healthy behavior change including more exercise and improved diet (13). Yet, there remains much to be understood about the interconnections between social capital, diabetes management, and hospital burdens. In countries such as Canada and Japan for example, weak social capital and sense of community belonging have been associated with health-related quality of life deficits and poor diabetes control among community-dwelling adults, and this despite universal healthcare coverage (2, 14). To our knowledge, no study has examined the relationship between community belonging and the risk of potentially avoidable hospitalization among adults living with diabetes. This investigation aims to overcome this evidence gap. In Canada, diabetes prevalence stood at 7.8% (males: 9.0%; females: 6.7%) in 2019, with the rate showing little sign of abating over time (15). Taking advantage of the innovative research avenues opened by linked household survey and healthcare administrative data sources (16, 17), we examine the role of individuals' sense of community belonging with the risk of hospitalization among the aging Canadian population living with diabetes.

## Materials and Methods

### Data Sources

This study drew on a unique resource of multiple years of data from the Canadian Community Health Survey (CCHS) linked to the hospital Discharge Abstract Database (DAD). Conducted annually by Statistics Canada, the CCHS collects a wide range of demographic and health data from a nationally representative sample of ~65,000 household respondents using a multistage stratified cluster sampling design. Survey weights are calculated by the national statistics agency to adjust for the complex sampling frame and interview non-responses (e.g., the 2010 person-level response rate was 89%) (18). The DAD collates demographic, administrative, and diagnostic data by fiscal year for all acute-care hospitalizations in 12 of the country's 13 jurisdictions (excluding the province of Quebec). Given the Canadian context of single-payer universal coverage, the administrative health data are considered complete recordings. Based on a probabilistic microdata linkage process [described elsewhere (19, 20)], we pooled and linked 5 years of CCHS cycles (2007–2011) with 8 years of DAD datasets (2005/06–2012/13) to obtain sufficient sample sizes of diabetes-related admissions. An evaluation elsewhere reported the survey-inpatient data linkage coverage rates to be over 90% among both women and men (21).

### Measures

The study sample included survey respondents who indicated they had been told by a health professional they had diabetes mellitus, as identified according to the CCHS core module including a series of questions on chronic conditions (22). As with all population-based data, survey responses may be subject to measurement error. Statistical triangulations and algorithms applied to CCHS data elsewhere have suggested the likelihood of misclassifications to be relatively minor (22, 23), although the survey information have not been clinically validated to distinguish between types 1 and 2 diabetes (24). The present analysis was limited to those aged 45 and over, when diabetes-attributable hospitalizations gain in relative frequency (17).

The outcome of interest was whether the individual had been hospitalized at least once for diabetes during the period of observation, based on the DAD data. This included any record where the primary diagnosis for the length of stay was for type 1 or type 2 diabetes and its complications (e.g., diabetic retinopathy, nephropathy, or certain circulatory complications), as coded to the International Classification of Diseases, 10th revision (ICD-10-CA codes E10-E14) (25). Validation analyses of diabetes-related diagnostic coding in DAD data have indicated high sensitivity (81.5–92.1%), specificity (93.9–97.0%), and positive predictive values (81.4–90.5%) (26). Diabetes is generally considered as ambulatory care sensitive, that is, a condition for which the need for hospital admission can be largely prevented or delayed through community factors (27, 28).

The key hypothesized predictor variable was the strength of belonging to one's local community, as captured in the CCHS based on the question, “How would you describe your sense of belonging to your local community?” (3). Responses were dichotomized as strong (or somewhat strong) vs. weak (or somewhat weak). Other personal characteristics were considered as confounding factors including individuals' age, gender, education, and place of residence. We further controlled for daily vegetables and fruits consumption, as a tracer for behavioral factors widely associated with disparities in diabetes health outcomes (29).

### Statistical Analysis

Following a brief descriptive analysis of the study population, multiple logistic regression was used to tease the independent association of community belonging with the risk of diabetes hospitalization. Pooling estimates of multiple years of survey and administrative data were considered to reflect an average risk of potentially avoidable hospitalization among a combined population roughly corresponding with the 2006–2012 period of observation (30). Regression parameters were estimated applying bootstrapped survey weights, to account for the complex CCHS sampling methods and ensure population representation of the results (30). Descriptive unweighted counts were rounded and adjusted to reinforce the confidential nature of the data using Statistics Canada control algorithms. Individual characteristics at the time of the survey were assumed to represent those at the time of the hospital episode. The analysis was limited to respondents with valid information on all variables of interest. To ease interpretation, results are expressed in terms of odds ratios (ORs) and 95% confidence intervals (CIs), which were estimated using the Stata v15 statistical software.

### Research Ethics

The de-identified linkable datasets were accessed in the Statistics Canada Research Data Centre located at the University of New Brunswick, Canada, in accordance with data privacy and security protocols. This study complied with the University of New Brunswick's Research Ethics Board, which does not require an internal institutional review for research projects using data accessed through the Research Data Centre network.

## Results

Of the 270,210 survey respondents who agreed to share and link their data, 15,560 were aged 45 and over living with diabetes and residing in any of nine provinces or three territories (excluding Quebec). Among these, 13,580 (87.3%) reported valid information for all variables of interest. The derived sample was designed to represent 5.4 million person-years of living with diabetes over the period of observation.

**Figure 1:**
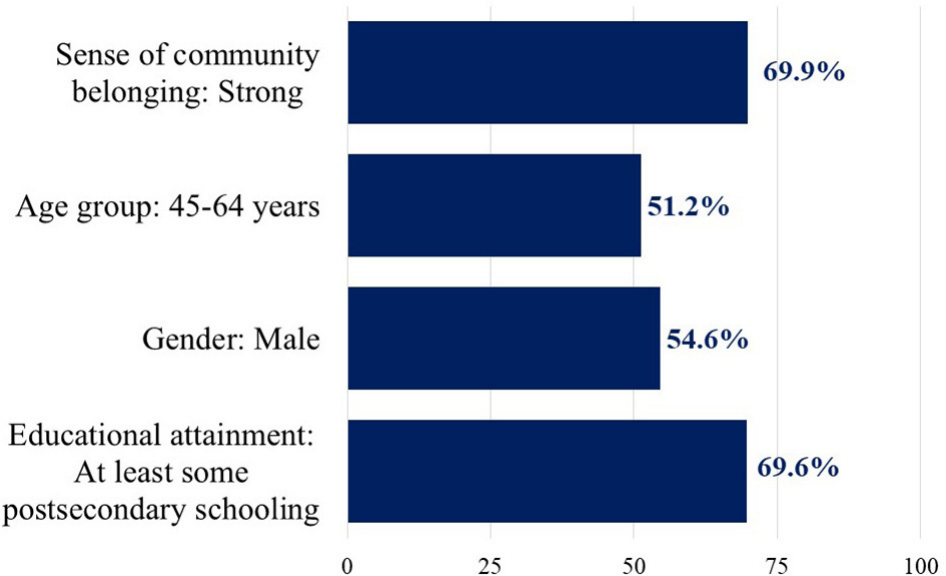
Percent of the Canadian population 45 years and over living with diabetes by selected characteristics. Data weighted for population representation. Source: Linked Canadian Community Health Survey—Discharge Abstract Database (*n* = 13,580).

Most (69.9%) of the aging adults with diabetes reported having a strong sense of belonging to their local community (Figure 1). Consistent with global epidemiological patterns, more males than females were living with diabetes (54.6 vs. 45.4%). Two-thirds (69.6%) had at least some post-secondary schooling.

Following data linkage, the rate of first diabetes-related hospital admissions was estimated at 9.5 per 1,000 person-years of the community-dwelling population 45 and over living with diabetes. A bivariate analysis revealed that those who reported weak community belonging were significantly more likely to have ever been hospitalized for diabetes and its complications compared with those reporting strong belonging (12.1 vs. 8.9 per 1,000 person-years) (*N* = 180; χ^2^ = 13.82; *p* < 0.05).

Based on the multiple regression analysis (Table 1), the association between weak community attachment and higher risk of diabetes hospitalization remained significant [OR: 1.80 (95% CI: 1.12–2.90)], after controlling for age, gender, education, place of residence, and healthy eating behaviors. Community residents in the 45–64 years category were significantly more likely to have been admitted for acute care than their older counterparts [OR: 1.76 (95% CI: 1.14–2.71)]. Rural residence was also significantly associated with higher hospitalization risk [OR: 1.79 (95% CI: 1.05–3.04)]. Results were suggestive, albeit not statistically convincing, of a protective influence of female gender and higher educational attainment.

**Table 1:** Adjusted odds ratios and 95% confidence intervals from the multiple logistic regression for the risk of diabetes hospitalization.

**Characteristic**	**Odds ratio**	**Confidence interval**		***p*-value**
		**Lower**	**Upper**	
**Sense of community belonging**				
Strong (ref)	1.00			
Weak	1.80^*^	1.12	2.90	0.015
**Age group**				
45–64 years	1.76^*^	1.14	2.71	0.011
65 years and over (ref)	1.00			
**Gender**				
Female	0.84	0.52	1.36	0.481
Male (ref)	1.00			
**Educational attainment**				
At most secondary graduation (ref)	1.00			
Any postsecondary schooling	0.67	0.41	1.08	0.099
**Place of residence**				
Rural	1.79^*^	1.05	3.04	0.032
Urban (ref)	1.00			
**Consumption of vegetables and fruits**				
Daily consumption (number)	0.98	0.88	1.10	0.781

* *p < 0.05; ref = reference category*.

*Source: Linked Canadian Community Health Survey—Discharge Abstract Database (n = 13,580)*.

## Discussion

A growing body of literature has pointed to various dimensions of social capital as vital to maintaining and improving population health, including in contexts of universal healthcare coverage (2, 3, 5, 6, 19). This study represents, to our knowledge, the first assessment of the role of community belonging among aging adults living with diabetes as a predictor of potentially avoidable hospitalization. While most (69.9%) Canadian adults aged 45 and over with diabetes reported having a strong sense of community belonging, the risk of admission for diabetes-related complications was found to be significantly higher among those with weak community ties [OR: 1.80 (95% CI: 1.12–2.90)], after adjusting for other sociodemographic and behavioral factors.

The present findings were consistent with research elsewhere on community belonging as a social determinant of different diabetes-related outcomes, using (unlinked) survey sources, including health utility deficits, glycemic control, and comorbidities (2, 14, 31). Increased community interactions may bolster health through the transmission of health promoting behaviors as a social norm, psychosocial mechanisms nurturing better self-rated health, and improved access to community resources; conversely, community disconnect may be syndemically damaging to mental and physical health (3, 13).

This study builds the evidence base on social capital among people living with diabetes by drawing on multiple years of nationally representative linked survey and administrative datasets to examine the risk of acute-care hospitalization, a statistically rare event but among the costliest categories to the healthcare system. The use of linked data offers analytical opportunities not afforded by a single source, notably the ability to account for individuals' diabetes status and their strength of belonging (since hospital records do not capture diagnoses in primary care or psychosocial variables) (16). Our results underscore the increasing emphasis of shifting the focus from acute care to supporting patients with chronic conditions through community-based interventions facilitating positive social networks, such as developing transportation networks, recreation programs and hobby groups, or cohousing communities for older adults (2, 6, 32–34). In the era of COVID-19, expanding access to digital communications may also help generate social capital (10).

Certain limitations to the analysis must be remarked including, firstly, the reliance on only one measure of social capital, that is, sense of community belonging, which may be perceived differently among survey respondents and across contexts (3, 7). Second is the risk of measurement error; 3.9% of the CCHS respondents were missing valid data on community belonging and thus were omitted. Third, due to sample size constraints (*N* = 180 acute-care admissions over the period of observation), we were not able to cover the full array of potential medical and non-medical predictors of adverse diabetes-related outcomes and hospitalization risks, such as adherence to self-management practices, presence and types of comorbid conditions, having a regular primary care practitioner, food insecurity, disability status, or living alone (6, 12, 16, 20, 24, 35). Fourth is the risk of reverse causality; it is possible that some respondents may have perceived and reported a weakening of social ties following prolonged or repeated hospitalizations.

## Conclusion

Global estimates indicate that diabetes prevalence quadrupled among adults worldwide in the last four decades (36). It has been postulated that low social capital may exacerbate existing challenges for diabetes management (29). Our study highlighted the costs of inaction by revealing a significantly increased risk of acute-care hospitalization with weak community belonging among aging adults with diabetes, even in the absence of direct financial barriers to accessing essential primary care services. The results add weight to the importance of considering not only biomedical risks but also social vulnerability for preventive public health measures to reduce the hospital burden of diabetes.

## Data Availability Statement

The datasets presented in this article are not readily available because data privacy and confidentiality are protected by the Canadian Statistics Act. Requests to access the datasets should be directed to Canadian Research Data Centre Network (crdcn.org/data).

## Ethics Statement

Ethical review and approval was not required for the study on human participants in accordance with the national legislation and institutional requirements.

## Author Contributions

NG conceptualized the study and led the writing of the manuscript. ZS performed the data management and formal analysis. All authors contributed to interpreting the results and read and approved the final manuscript.

## Conflict of Interest

The authors declare that the research was conducted in the absence of any commercial or financial relationships that could be construed as a potential conflict of interest.
